# The limits of personal experience

**DOI:** 10.3389/fpsyg.2024.1365180

**Published:** 2024-10-21

**Authors:** Venkat Ram Reddy Ganuthula

**Affiliations:** Indian Institute of Technology Jodhpur, Jodhpur, India

**Keywords:** personal experience, decision-making, rationality, information, experience

## Abstract

This article examines how three types of experience—personal, related others, and unrelated others—influence decision-making. We present the complexities and nuances in using these experiential sources to suggest that personal experience is preferred to the other two sources. We discuss the implications of this preference for decision-making processes, especially in contexts involving transformative outcomes. To conclude, we discuss how people rely on other experiential sources when their preferred source is limited.

## Introduction

Humans are goal-oriented creatures (Aarts and Elliot, 2012). They interpret their surroundings subjectively and use them to achieve their goals. However, environments usually provide people with more than one action option to achieve the desired results (Pezzulo et al., 2014). People weigh competing options to select those that will best help them achieve their objectives (Friedrich and Lengyel, 2016). However, this seemingly simple dynamic of decision-making is fraught with complications. These difficulties stem from the fact that the outcomes of the action alternatives occur in the future, emphasizing the role of uncertainty and chance. To navigate uncertainties and make choices, people use their cognitive resources to gather and process information (Simon, 1955; Evans, 2008). In decision-making contexts, two primary types of information are utilized: descriptive and experiential (Hertwig and Erev, 2009).

Descriptive information encompasses the immediate availability of sensory stimuli, representations of action alternatives, desired outcomes, and the specific behavioral context (Pezzulo et al., 2014). This information is easily accessible in many decision-making situations and assists individuals in the process of sensemaking, thereby facilitating an understanding of both known and unknown factors (Hertwig et al., 2018). In situations where clear data or statistics are available, descriptive information can be particularly valuable in evaluating both short-term and long-term risks. For instance, descriptive information, including market trends, company financial reports, and economic indicators, is essential for investors to make informed decisions in financial decision-making (Barberis and Thaler, 2003).

Conversely, experiential information refers to knowledge and understanding acquired through events that transcend time and space. This assists individuals in assessing their past behaviors and directing their future actions by offering them a sense of what could and could not be effective in a decision-making context (Hertwig and Erev, 2009; March, 2010; Kolb, 2014). Complex cognitive mechanisms of memory consolidation and retrieval are involved in this process (Nadel and Moscovitch, 1997). For example, an individual’s prior experiences in various work environments or with a variety of responsibilities can have a substantial impact on their future career decisions (Super, 1980; Seibert et al., 2024).

This paper primarily focuses on experiential information, investigating its subtleties and influence on decision-making processes. In particular, we will discuss three sources of experiential information: personal experience, the experience of related others, and the experience of unrelated others. We posit that decision-makers employ a hierarchical approach when seeking experiential information, as outlined in the social-circle heuristic concept introduced by Pachur et al. (2013). Initially, they depend on personal experience as the primary source. If their personal experience is inadequate, they consult the experiences of known others (related others). In the event that the information provided by known individuals is still insufficient, they will then evaluate the experiences of acquaintances or experts. This hierarchical approach is indicative of the natural progression from more immediate and reliable sources of information to broader, potentially less reliable, but more comprehensive sources.

In the following sections, we will discuss research on the use of personal experience before moving on to discuss how people utilize the experiences of related others and unrelated others when personal experience proves insufficient. By delving into these aspects, we aim to shed light on how individuals specifically navigate transformative decision contexts that fundamentally change the decision-maker’s life in ways they cannot fully anticipate beforehand.

## The personal experience

Personal experience, along with intuition—often informed by such experience—is the most readily accessible source of experiential information among the three sources previously mentioned (Reed, 1996). People generally trust their personal experience, considering it a primary and useful source of experiential information for decision-making in a variety of contexts (Myers, 2007), including the making of scientific discoveries and the conduct of themselves in everyday situations (Hogarth, 2010). Although there has been considerable discourse regarding the usefulness of personal experience in decision-making (see Hogarth (2010) for a comprehensive examination), reservations regarding its efficacy are not uncommon.

Classic work by Ross et al. (1977) introduced the concept of the false-consensus effect, highlighting how people tend to overestimate the extent to which others share their beliefs and attitudes. These tendencies to overgeneralize personal experiences beyond their actual relevance significantly impact decision-making. However, Dawes (1989) argues that people’s notions about shared beliefs and attitudes could stem from valid generalizations of one’s own experiences.

More recent research suggests that people tend to process information from the standpoint of their ego (Epley et al., 2004). While, as described by the false-consensus effect, people view their own characteristics as more common than they actually are, egocentric processing of information biases people to attend to those experiences that conform to their current beliefs and desires (Epley et al., 2006). Thus, these processes raise more questions about the utility of personal experience in decision-making.

The extensive evidence on how intuitive processing of information can influence decision-making is provided by the research tradition on heuristics and biases (Tversky and Kahneman, 1974). This body of knowledge implies that individuals employ cognitive shortcuts to draw upon their personal experiences when making decisions. For instance, the availability heuristic posits that individuals typically evaluate the probability of an event by assessing the ease with which they can recall relevant examples from their personal experiences (Tversky and Kahneman, 1973).

Heuristics frequently prioritize personal experience, such as the availability heuristic, which can result in biases. For example, the representativeness heuristic may lead individuals to disregard base rates in favor of more vivid personal experiences (Kahneman and Tversky, 1972). Similarly, the affect heuristic can cause individuals to make judgments based on emotional responses to past experiences, potentially disregarding other pertinent information (Slovic et al., 2007).

While heuristics can occasionally lead to biases, they are frequently effective decision-making tools, particularly in situations where they are consistent with the structure of the information (Gigerenzer and Gaissmaier, 2011). The critical aspect is comprehending the contexts in which these heuristics are implemented and the manner in which they interact with personal experience in a variety of decision-making scenarios.

Overall, while personal experience assumes primacy as a source of information for decision-making, it has limitations, as deliberated above. People frequently encounter situations where their own experience is limited or impertinent (Dunbar, 1998). Realizing that they do not operate in isolation, individuals frequently turn to the experiences of others in their social milieu to mitigate uncertainty and make more informed decisions (Henrich, 2015). In the next section, we’ll explore how people build on the experiences of their social networks to handle complex decision-making landscapes.

## The experience of related others

Individuals often rely on the experiences of others in their social networks to make decisions. This dependence on related individuals is prevalent in a wide range of situations, from everyday choices to specialized professional contexts. For example, apprentice physicians acquire the ability to accurately identify tissue specimens from their mentors (Puskaric et al., 2018). Through such sharing of experiences, individuals are able to make more informed decisions and achieve their objectives (Boyd et al., 2011).

Research on “social sampling” offers a comprehensive understanding of the manner in which individuals employ both personal and social information in their decision-making processes (Hertwig et al., 2005; Galesic et al., 2018; Pachur et al., 2013; Schulze et al., 2021). These studies investigate a variety of social sampling components, such as the manner in which individuals access and utilize personal and others’ experiences, assess their validity, and how these factors affect decision-making. For instance, Galesic et al. (2018) propose that individuals may infer characteristics of the broader population from their narrow social networks, which can result in biases in the processing of social information.

A recent study conducted by Schulze et al. (2021) offers valuable insights into the individual differences that occur in social sampling. Their research illustrates that individuals differ in their degree of dependence on various social subgroups when formulating assessments. This finding underscores the intricacy of social sampling procedures and implies that the application of social information in decision-making is not consistent across individuals or circumstances.

In a similar vein, research on meta-cognitive myopia, a cognitive bias that posits that individuals place a high value on immediate available information while disregarding broader contexts, provides further insights (Fiedler, 2000; Fiedler et al., 2023). This research posits that the quality of decisions can be influenced by the fact that individuals frequently lack a comprehensive understanding of the historical validity and nature of the social samples they employ. Additionally, research indicates that individuals’ decision-making processes are influenced by factors such as the reputation and status of the source in the referent social networks (McDonald and Westphal, 2003; Agneessens and Wittek, 2012).

In addition to the experiences of individuals in their immediate social circles, individuals also acquire knowledge from the broader social norms that influence collective behaviors in their immediate social networks (Tankard and Paluck, 2016). Social norms provide an implicit comprehension of what is appropriate in a social context; however, they can become dysfunctional if they fail to adapt to changing environmental contexts (Bicchieri, 2016). So, in order to avoid bad results, it is necessary to strike a balance between the information that individuals acquire from their peers and the relevance of this information to the specific decision context.

In sum, learning from related others’ experience helps reduce uncertainty and allows for making better decisions, particularly when personal experience is costly or difficult to source (Franz and Matthews, 2010; Grüter et al., 2010). However, using social information for decision-making is fraught with issues such as the appropriateness of weighting, meta-cognitive limitations, and the existence of maladaptive social norms. To overcome these concerns, decision-makers may rely on another layer of experiential information from those unrelated to them (Weizsäcker, 2010; Yaniv and Kleinberger, 2000). In the next section, we’ll explore how people acquire and apply experiential information from acquaintances and experts.

## The experience of unrelated others

People often turn to the experiences of acquaintances, including friends, colleagues, and casual contacts, to inform their decision-making when personal experience is limited. The acquisition of information from these sources is influenced by several factors, including the structure of one’s social network. The concept of “weak ties” suggests that casual acquaintances often provide novel information not available within one’s immediate social circle, potentially leading to more informed decisions (Granovetter, 1973; Park et al., 2018; Rajkumar et al., 2022).

However, the tendency for individuals to associate with similar others, known as homophily, can affect the diversity of experiential information received (McPherson et al., 2001; Ertug et al., 2022). While this can lead to more relatable experiences, it may also limit exposure to diverse perspectives and experiences. Further, the perceived trustworthiness of acquaintances affects the weight given to their experiences (Levin and Cross, 2004; Kim and Fernandez, 2023). This can lead to information cascades, where individuals adopt behaviors based on others’ actions (Bikhchandani et al., 1992), potentially resulting in beneficial knowledge sharing or problematic groupthink (Tokita et al., 2021).

While acquaintances provide one source of experiential information, individuals also frequently seek advice from experts. Experts contribute valuable experiential knowledge gained from years of training and practice (Ericsson and Lehmann, 1996). For example, individuals often consult professional forecasters or business consultants for specialized insights (Nikolova, 2007; Nibbering et al., 2018).

The impact of expert advice, however, can be influenced by factors beyond the expert’s actual expertise. One such factor is the perceived psychological distance between decision-makers and experts (Blunden et al., 2019). This phenomenon is related to egocentric advice discounting, where individuals tend to undervalue others’ advice compared to their own opinions (Yaniv and Kleinberger, 2000).

The credibility and trustworthiness of experts have a substantial impact on the perceived value of their advice (Sniezek and Van Swol, 2001). It is also important to consider the context in which expert advice is sought. In conditions of significant uncertainty, individuals may place a greater emphasis on the opinions of experts (Bonaccio and Dalal, 2006). Nevertheless, in areas where individuals perceive themselves as competent, they may combine their own judgments with expert advice (Gino and Moore, 2007).

By leveraging experiences from both acquaintances and experts, decision-makers can access a broader pool of experiential information. However, effective use of this information requires careful consideration of various factors influencing its acquisition, interpretation, and application in decision-making processes. Understanding these dynamics can help individuals make more informed choices by optimally integrating diverse sources of experiential knowledge.

In sum, we have examined the relative benefits and challenges associated with the use of three sources of experiential information: personal, related others, and experts. While the experiences of related others and experts can provide valuable insights, they are often sought as supplements or alternatives to personal experience. However, there are certain decision contexts where even these additional sources of information may prove inadequate. This is particularly true for what we term ‘transformative decisions’ - choices that fundamentally alter one’s life in ways that cannot be fully anticipated. The following section will explore these limitations in depth, focusing on how the nature of transformative decisions creates unique challenges for decision-makers.

## The limits of personal experience and decision-making

Decision-making is a complex process drawing on various information sources. The social circle heuristic suggests individuals first turn to personal experience, then to related others, and finally to acquaintances and experts (Pachur et al., 2013). This preference is influenced by factors such as information availability, access costs, and decision context predictability (Laland, 2004). However, in the context of transformative decisions, this heuristic faces significant challenges.

Paul (2014, 2015) on ‘transformative decisions’ highlights choices that fundamentally change the decision-maker’s life in unpredictable ways. These decisions are characterized by novelty, high uncertainty (Hogarth et al., 2015; Paul, 2014), and often irreversible outcomes (Fischhoff, 2015; Taleb, 2007; Hammond et al., 1998). They commit the decision-maker to a particular path with lasting implications (Gilbert and Ebert, 2002).

A prime example is choosing a career path, which involves significant uncertainty about future job markets, personal fit, and long-term satisfaction (Super, 1980). For instance, a student deciding whether to pursue a career in artificial intelligence faces uncertainty about the field’s future developments, their own aptitude for the work, and the long-term societal implications of AI (Brynjolfsson and Mitchell, 2017). The transformative nature of this decision lies in its potential to shape one’s identity, social network, and life trajectory in profound and often unpredictable ways.

In contrast, many mundane decisions are rooted in explicit outcome representations and immediate hedonic rewards (Hoffman and Nordgren, 2015). These decisions are often reversible and insignificant (Gilbert and Ebert, 2002). For example, it’s relatively simple to discard an unsatisfactory meal or switch to a preferred drink.

When making transformative decisions, individuals lack personal experience, which is typically the most reliable and preferred source of information (Hertwig and Erev, 2009). This reveals a peculiar human condition: people are deprived of their preferred information source when making the most significant decisions that shape their lives. Consequently, achieving optimal outcomes in these critical situations becomes exceedingly difficult (Gilbert and Wilson, 2007; Schwartz, 2004). This limitation of personal experience in crucial decision-making contexts presents a significant challenge for human decision-making and well-being, underscoring the complexity of navigating life’s most impactful choices.

In the absence of personal experience, people often turn to the experiences of related and unrelated others to make transformative choices (Yaniv, 2004). In some cases, individuals can efficiently incorporate others’ experiences through simple heuristics or social learning mechanisms (Hertwig et al., 2012; Rendell et al., 2010). For example, they might use the “imitate-the-successful” heuristic, where they copy the behavior of individuals who appear to be thriving, or employ social learning strategies like conformity bias, where they adopt the most common behavior in their social group.

However, effectively using others’ experiences often requires additional cognitive effort, particularly in complex or unfamiliar situations (Yaniv, 2004). This process involves assessing the relevance and applicability of others’ experiences (Medin et al., 2003), integrating potentially conflicting information (Bonaccio and Dalal, 2006), evaluating source credibility (Birnbaum and Stegner, 1979), and adapting insights to one’s unique circumstances (Bandura, 1977).

To navigate this complexity, individuals employ various cognitive strategies that, while potentially adding some cognitive load, ultimately serve to simplify and structure the decision-making process. These strategies include selectively filtering information aligning with existing beliefs (Nickerson, 1998), using analogical reasoning to apply insights from one context to another (Gentner and Smith, 2012), relying on social proof in uncertain situations (Cialdini, 2009), engaging in adaptive learning to refine their approach over time (Erev and Roth, 2014), and regulating emotional responses to maintain a balanced perspective (Gross, 2015). These processes help individuals organize and interpret the diverse array of information available from others’ experiences. The effort invested in applying these strategies often corresponds to the perceived importance of the decision (Loewenstein and Schkade, 1999).

For example, an individual considering whether to pursue a PhD program might employ these strategies by seeking out and focusing on stories from successful graduates (information filtering), comparing challenges described by current students to their own past academic experiences (analogical reasoning), examining statistics on completion rates and career outcomes (social proof), adjusting their expectations as they gather more information (adaptive learning), and managing their anxiety or excitement about the potential decision by reflecting on diverse experiences encountered (emotional regulation).

This multifaceted approach to integrating others’ experiences into one’s own decision-making underscores the nuanced and cognitively demanding nature of learning from others and effectively applying that knowledge to one’s own circumstances. It illustrates how individuals compensate for the lack of personal experience in transformative decisions by leveraging a complex interplay of cognitive strategies and social learning mechanisms.

## Coda

In conclusion, while personal experience is the preferred source of information for decision-making, its absence in transformative decisions forces individuals to rely on the experiences of others. This process involves complex cognitive strategies and social learning mechanisms, highlighting the challenges and intricacies of making life-altering decisions without direct personal experience.

## Data Availability

The original contributions presented in the study are included in the article/supplementary material, further inquiries can be directed to the corresponding author.

## References

[ref1] Aarts H. Elliot A. (2012). Goal-directed behavior. London: Psychology Press.

[ref2] Agneessens F. Wittek R. (2012). Where do intra-organizational advice relations come from? The role of informal status and social capital in social exchange. Soc. Networks 34, 333–345. doi: 10.1016/j.socnet.2011.04.002

[ref3] Bandura A. (1977). Social learning theory. Englewood Cliffs, NJ: Prentice Hall.

[ref4] Barberis N. Thaler R. (2003). “A survey of behavioral finance” in Handbook of the economics of finance. eds. Constantinides G. M. Harris M. Stulz R. M.. 1st ed (Amsterdam: Elsevier), 1053–1128.

[ref5] Bicchieri C. (2016). Norms in the wild: How to diagnose, measure, and change social norms. New York: Oxford University Press.

[ref6] Bikhchandani S. Hirshleifer D. Welch I. (1992). A theory of fads, fashion, custom, and cultural change as informational cascades. J. Polit. Econ. 100, 992–1026. doi: 10.1086/261849

[ref7] Birnbaum M. H. Stegner S. E. (1979). Source credibility in social judgment: Bias, expertise, and the judge's point of view. J. Pers. Soc. Psychol. 37, 48–74. doi: 10.1037/0022-3514.37.1.48

[ref8] Blunden H. Logg J. M. Brooks A. W. John L. K. Gino F. (2019). Seeker beware: the interpersonal costs of ignoring advice. Organ. Behav. Hum. Decis. Process. 150, 83–100. doi: 10.1016/j.obhdp.2018.12.002

[ref9] Bonaccio S. Dalal R. S. (2006). Advice taking and decision-making: an integrative literature review and implications for the organizational sciences. Organ. Behav. Hum. Decis. Process. 101, 127–151. doi: 10.1016/j.obhdp.2006.07.001

[ref10] Boyd R. Richerson P. J. Henrich J. (2011). The cultural niche: why social learning is essential for human adaptation. Proc. Natl. Acad. Sci. 108, 10918–10925. doi: 10.1073/pnas.1100290108, PMID: 21690340 PMC3131818

[ref9001] Brynjolfsson E. Mitchell T., (2017). What can machine learning do? Workforce implications. Science, 358, 1530–1534. 29269459 10.1126/science.aap8062

[ref12] Cialdini R. B. (2009). Influence: Science and practice. 5th Edn. Boston: Allyn & Bacon.

[ref13] Dawes R. M. (1989). Statistical criteria for establishing a truly false consensus effect. J. Exp. Soc. Psychol. 25, 1–17. doi: 10.1016/0022-1031(89)90036-X

[ref15] Dunbar R. I. M. (1998). The social brain hypothesis. Evolutionary Anthropol. 6, 178–190. doi: 10.1002/(SICI)1520-6505(1998)6:5<178:AID-EVAN5>3.0.CO;2-8

[ref16] Epley N. Caruso E. M. Bazerman M. H. (2006). When perspective taking increases taking: reactive egoism in social interaction. J. Pers. Soc. Psychol. 91, 872–889. doi: 10.1037/0022-3514.91.5.872, PMID: 17059307

[ref17] Epley N. Keysar B. Van Boven L. Gilovich T. (2004). Perspective taking as egocentric anchoring and adjustment. J. Pers. Soc. Psychol. 87, 327–339. doi: 10.1037/0022-3514.87.3.327, PMID: 15382983

[ref18] Ericsson K. A. Lehmann A. C. (1996). Expert and exceptional performance: evidence of maximal adaptation to task constraints. Annu. Rev. Psychol. 47, 273–305. doi: 10.1146/annurev.psych.47.1.273, PMID: 15012483

[ref9002] Erev I. Roth A. E., (2014). Maximization, learning, and economic behavior. Proceedings of the National Academy of Sciences, 111(Supplement 3), 10818–10825. 10.1073/pnas.1402846111 PMC4113920 25024182

[ref9003] Ertug G. Brennecke J. Kovács B. Zou T., (2022). What does homophily do? A review of the consequences of homophily. Acad Manag Ann. 16, 38–69.

[ref19] Evans J. S. B. (2008). Dual-processing accounts of reasoning, judgment, and social cognition. Annu. Rev. Psychol. 59, 255–278. doi: 10.1146/annurev.psych.59.103006.093629, PMID: 18154502

[ref20] Fiedler K. (2000). Beware of samples! A cognitive-ecological sampling approach to judgment biases. Psychol. Rev. 107, 659–676. doi: 10.1037/0033-295X.107.4.659, PMID: 11089402

[ref21] Fiedler K. Prager J. McCaughey L. (2023). Meta-cognitive myopia: a pervasive, often implicit fallacy in human judgment and decision making. Curr. Dir. Psychol. Sci. 32, 49–56. doi: 10.1177/09637214221126906

[ref22] Fischhoff B. (2015). The realities of risk-cost-benefit analysis. Science 350:aaa6516. doi: 10.1126/science.aaa6516, PMID: 26516286

[ref23] Franz M. Matthews L. J. (2010). Social enhancement can create adaptive, arbitrary, and maladaptive cultural traditions. Proc. R. Soc. Lond. B Biol. Sci. 277, 3363–3372. doi: 10.1098/rspb.2010.0705, PMID: 20547762 PMC2981925

[ref24] Friedrich J. Lengyel M. (2016). Goal-directed decision making with spiking neurons. J. Neurosci. 36, 1529–1546. doi: 10.1523/JNEUROSCI.2854-15.2016, PMID: 26843636 PMC4737768

[ref26] Galesic M. Olsson H. Rieskamp J. (2018). A sampling model of social judgment. Psychol. Rev. 125, 363–390. doi: 10.1037/rev0000096 29733664

[ref28] Gentner D. Smith L. (2012). “Analogical reasoning” in Encyclopedia of human behavior. ed. Ramachandran V. S.. 2nd ed (Oxford: Elsevier), 130–136.

[ref29] Gigerenzer G. Gaissmaier W. (2011). Heuristic decision making. Annu. Rev. Psychol. 62, 451–482. doi: 10.1146/annurev-psych-120709-145346 21126183

[ref30] Gilbert D. T. Ebert J. E. (2002). Decisions and revisions: the affective forecasting of changeable outcomes. J. Pers. Soc. Psychol. 82, 503–514. doi: 10.1037/0022-3514.82.4.503, PMID: 11999920

[ref31] Gilbert D. T. Wilson T. D. (2007). Prospection: experiencing the future. Science 317, 1351–1354. doi: 10.1126/science.1144161 17823345

[ref32] Gino F. Moore D. A. (2007). Effects of task difficulty on use of advice. J. Behav. Decis. Mak. 20, 21–35. doi: 10.1002/bdm.539

[ref33] Granovetter M. S. (1973). The strength of weak ties. Am. J. Sociol. 78, 1360–1380. doi: 10.1086/225469

[ref34] Gross J. J. (2015). Emotion regulation: current status and future prospects. Psychol. Inq. 26, 1–26. doi: 10.1080/1047840X.2014.940781

[ref35] Grüter C. Leadbeater E. Ratnieks F. L. W. (2010). Social learning: the importance of copying others. Curr. Biol. 20, R683–R685. doi: 10.1016/j.cub.2010.06.052, PMID: 20728057

[ref36] Hammond J. S. Keeney R. L. Raiffa H. (1998). The hidden traps in decision making. Harv. Bus. Rev. 76:47, PMID: 10185432

[ref37] Henrich J. (2015). The secret of our success: How culture is driving human evolution, domesticating our species, and making us smarter. Princeton, NJ: Princeton University Press.

[ref38] Hertwig R. Erev I. (2009). The description-experience gap in risky choice. Trends Cogn. Sci. 13, 517–523. doi: 10.1016/j.tics.2009.09.004, PMID: 19836292

[ref39] Hertwig R. Hoffrage U. The ABC Research Group (2012). Simple heuristics in a social world. Oxford: Oxford University Press.

[ref40] Hertwig R. Hogarth R. M. Lejarraga T. (2018). Experience and description: exploring two paths to knowledge. Curr. Dir. Psychol. Sci. 27, 123–128. doi: 10.1177/0963721417740645

[ref41] Hertwig R. Pachur T. Kurzenhäuser S. (2005). Judgments of risk frequencies: tests of possible cognitive mechanisms. J. Exp. Psychol. Learn. Mem. Cogn. 31, 621–642. doi: 10.1037/0278-7393.31.4.621, PMID: 16060769

[ref42] Hoffman W. M. Nordgren L. F. (2015). The psychology of desire. New York: Guilford Publications.

[ref43] Hogarth R. M. (2010). Intuition: a challenge for psychological research on decision making. Psychol. Inq. 21, 338–353. doi: 10.1080/1047840X.2010.520260

[ref44] Hogarth R. M. Lejarraga T. Soyer E. (2015). The two settings of kind and wicked learning environments. Curr. Dir. Psychol. Sci. 24, 379–385. doi: 10.1177/0963721415591878

[ref45] Kahneman D. Tversky A. (1972). Subjective probability: a judgment of representativeness. Cogn. Psychol. 3, 430–454. doi: 10.1016/0010-0285(72)90016-3

[ref9006] Kim M. Fernandez R. M., (2023). What makes weak ties strong?. Annual Review of Sociology, 49, 177–193. doi: 10.1146/annurev-soc-030921-034152

[ref46] Kolb D. A. (2014). Experiential learning: Experience as the source of learning and development. New Jersey: FT Press.

[ref47] Laland K. N. (2004). Social learning strategies. Anim. Learn. Behav. 32, 4–14. doi: 10.3758/BF03196002 15161136

[ref9004] Levin D. Z. Cross R., (2004). The strength of weak ties you can trust: The mediating role of trust in effective knowledge transfer. Manage Sci, 50, 1477–1490.

[ref48] Loewenstein G. Schkade D. (1999). “Wouldn't it be nice? Predicting future feelings” in Well-being: The foundations of hedonic psychology. eds. Kahneman D. Diener E. Schwarz N. (New York: Russell Sage Foundation), 85–105.

[ref49] March J. G. (2010). The ambiguities of experience. Ithaca, NY: Cornell University Press.

[ref50] McDonald M. L. Westphal J. D. (2003). Getting by with the advice of their friends: CEOs' advice networks and firms' strategic responses to poor performance. Adm. Sci. Q. 48, 1–32. doi: 10.2307/3556617

[ref51] McPherson M. Smith-Lovin L. Cook J. M. (2001). Birds of a feather: Homophily in social networks. Annu. Rev. Sociol. 27, 415–444. doi: 10.1146/annurev.soc.27.1.415

[ref52] Medin D. L. Ross N. O. Cox D. G. (2003). Culture and resource conflict: Why meanings matter. New York: Russell Sage Foundation.

[ref53] Myers D. G. (2007). The powers and perils of intuition. Sci. Am. Mind 18, 24–31. doi: 10.1038/scientificamericanmind0607-24

[ref54] Nadel L. Moscovitch M. (1997). Memory consolidation, retrograde amnesia, and the hippocampal complex. Curr. Opin. Neurobiol. 7, 217–227. doi: 10.1016/S0959-4388(97)80010-4, PMID: 9142752

[ref55] Nibbering D. Paap R. van der Wel M. (2018). What do professional forecasters actually predict? Int. J. Forecast. 34, 288–311. doi: 10.1016/j.ijforecast.2017.12.004

[ref56] Nickerson R. S. (1998). Confirmation bias: a ubiquitous phenomenon in many guises. Rev. Gen. Psychol. 2, 175–220. doi: 10.1037/1089-2680.2.2.175

[ref57] Nikolova N. (2007). The client-consultant relationship in professional business service firms. Cologne: Kölner Wissenschaftsverlag.

[ref58] Pachur T. Hertwig R. Rieskamp J. (2013). Intuitive judgments of social statistics: how exhaustive does sampling need to be? J. Exp. Soc. Psychol. 49, 1059–1077. doi: 10.1016/j.jesp.2013.07.004

[ref59] Park J. Newman M. E. J. Barabási A. L. (2018). The structure and dynamics of multilayer networks. Cambridge: Cambridge University Press.

[ref60] Paul L. A. (2014). Transformative experience. Oxford: Oxford University Press.

[ref61] Paul L. A. (2015). What you can't expect when you're expecting. Res Philosophica 92, 149–170. doi: 10.11612/resphil.2015.92.2.1

[ref62] Pezzulo G. Verschure P. F. Balkenius C. Pennartz C. (2014). The principles of goal-directed decision-making: from neural mechanisms to computation and robotics. Philos. Trans. R. Soc. B 369:20130470. doi: 10.1098/rstb.2013.0470, PMID: 25267813 PMC4186224

[ref63] Puskaric M. von Helversen B. Rieskamp J., (2018). How social information affects information search and choice in probabilistic inferences. A cta psychologica, 182, pp.166–176. doi: 10.1016/j.actpsy.2017.08.004 29179021

[ref64] Rajkumar K. Saint-Jacques G. Bojinov I. Brynjolfsson E. Aral S. (2022). A causal test of the strength of weak ties. Science 377, 1304–1310. doi: 10.1126/science.abl4476 36107999

[ref65] Reed E. S. (1996). The necessity of experience. New Haven, CT: Yale University Press.

[ref66] Rendell L. Boyd R. Cownden D. Enquist M. Eriksson K. Feldman M. W.. (2010). Why copy others? Insights from the social learning strategies tournament. Science 328, 208–213. doi: 10.1126/science.1184719, PMID: 20378813 PMC2989663

[ref67] Ross L. Greene D. House P. (1977). The "false consensus effect": an egocentric bias in social perception and attribution processes. J. Exp. Soc. Psychol. 13, 279–301. doi: 10.1016/0022-1031(77)90049-X

[ref68] Schulze C. Hertwig R. Pachur T. (2021). Who you know is what you know: modeling boundedly rational social sampling. J. Exp. Psychol. Gen. 150, 221–241. doi: 10.1037/xge0000799, PMID: 32915018

[ref69] Schwartz B. (2004). The paradox of choice: Why more is less. New York: Harper Collins.

[ref70] Seibert S. Akkermans J. Liu C. H. (2024). Understanding contemporary career success: a critical review. Annu. Rev. Organ. Psych. Organ. Behav. 11, 509–534. doi: 10.1146/annurev-orgpsych-120920-051543

[ref71] Simon H. A. (1955). A behavioral model of rational choice. Q. J. Econ. 69, 99–118. doi: 10.2307/1884852

[ref72] Slovic P. Finucane M. L. Peters E. MacGregor D. G. (2007). The affect heuristic. Eur. J. Oper. Res. 177, 1333–1352. doi: 10.1016/j.ejor.2005.04.006

[ref73] Sniezek J. A. Van Swol L. M. (2001). Trust, confidence, and expertise in a judge-advisor system. Organ. Behav. Hum. Decis. Process. 84, 288–307. doi: 10.1006/obhd.2000.2926, PMID: 11277673

[ref74] Super D. E. (1980). A life-span, life-space approach to career development. J. Vocat. Behav. 16, 282–298. doi: 10.1016/0001-8791(80)90056-1

[ref75] Taleb N. N. (2007). The black swan: The impact of the highly improbable. New York: Random House.

[ref76] Tankard M. E. Paluck E. L. (2016). Norm perception as a vehicle for social change. Soc. Issues Policy Rev. 10, 181–211. doi: 10.1111/sipr.12022

[ref78] Tokita C. K. Guess A. Tarnita C. E. (2021). Polarized information ecosystems can reorganize social networks via information cascades. Proc. Natl. Acad. Sci. 118:e2102147118. doi: 10.1073/pnas.2102147118, PMID: 34876511 PMC8685718

[ref79] Tversky A. Kahneman D. (1973). Availability: a heuristic for judging frequency and probability. Cogn. Psychol. 5, 207–232. doi: 10.1016/0010-0285(73)90033-9

[ref80] Tversky A. Kahneman D. (1974). Judgment under uncertainty: heuristics and biases. Science 185, 1124–1131. doi: 10.1126/science.185.4157.1124 17835457

[ref82] Weizsäcker G. (2010). Do we follow others when we should? A simple test of rational expectations. Am. Econ. Rev. 100, 2340–2360. doi: 10.1257/aer.100.5.2340

[ref84] Yaniv I. (2004). Receiving other people's advice: influence and benefit. Organ. Behav. Hum. Decis. Process. 93, 1–13. doi: 10.1016/j.obhdp.2003.08.002

[ref85] Yaniv I. Kleinberger E. (2000). Advice taking in decision making: egocentric discounting and reputation formation. Organ. Behav. Hum. Decis. Process. 83, 260–281. doi: 10.1006/obhd.2000.2909, PMID: 11056071

